# First 20 Years of Orbitrap Mass Spectrometry as the Mainstream Analytical Technique

**DOI:** 10.1002/mas.70024

**Published:** 2026-04-08

**Authors:** Alexander Makarov

**Affiliations:** ^1^ Thermo Fisher Scientific (Bremen) GmbH Bremen Germany

## Abstract

This review traces the first 20 years of Orbitrap mass spectrometry as a mainstream high‑resolution and accurate‑mass (HR/AM) technology. It outlines the historical development of the Orbitrap analyzer, the evolution of major instrument families, and the key technological innovations that enabled its widespread adoption. Particular emphasis is placed on hybrid and Tribrid architectures, democratization of HR/AM through benchtop platforms, extension to high‑mass and native analysis, and integration with ion mobility, advanced fragmentation methods, and emerging applications such as structural biology, isotope ratio measurements, and space research. The review concludes with a perspective on future directions and the anticipated role of Orbitrap instrumentation as the core workhorse in analytical laboratories worldwide.

**Abbreviations**
ADMET: absorption, distribution, metabolism, excretion, and toxicity
AGC: automatic gain control
AIF: all‑ion fragmentation
AP: atmospheric pressure
APPI: atmospheric‑pressure photoIonization
ASAP: atmospheric solids analysis probe
Astral: ASymmetric TRAck Lossless (analyzer)
CCS: collision cross‐section(s)
CDMS: charge detection mass spectrometry
CI: chemical Ionization
CID: collision‑induced dissociation
CryoEM: cryogenic electron microscopy
DART: direct analysis in real time
DDA: data‑dependent acquisition
DESI: desorption electrospray ionization
DIA: data‑independent acquisition
DMT: direct mass technology
ECD: electron‐capture dissociation
EDR: enhanced dynamic range
eFT: Enhanced Fourier Transform
EI: electron impact
ETciD: electron‑transfer/collision‑induced dissociation
ETD: electron transfer dissociation
EThcD: electron‑transfer/higher‑energy collision dissociation
FAIMS: field asymmetric waveform ion mobility spectrometry
FSDM: phase‑constrained signal deconvolution method
FT ICR: fourier transform ion cyclotron resonance
GC/MS: gas chromatography–mass spectrometry
HCD: higher‑energy collision dissociation
HCTT: high‐capacity transfer tube
HR/AM: high resolution and accurate mass
ICD: independent charge detector
ICP: inductively coupled plasma
IMS: ion mobility spectrometry
IRM: ion routing multipole
IRMPD: infrared multiphoton dissociation
LC: liquid chromatography
LDTD: laser diode thermal desorption
LIFDI: liquid injection field desorption ionization
LS‐APGD: liquid sampling–atmospheric‐pressure glow discharge
MALDESI: matrix‐assisted laser desorption electrospray ionization
MALDI: matrix‑assisted laser desorption/ionization
MAM: multi‑attribute method
MION: multi‐scheme chemical ionization
MS: mass spectrometry or mass spectrometer
OLED: organic light‐emitting diode(s)
OTMS: orbitrap mass spectrometry
PCB: printed circuit board
PFAS: per‐ and polyfluoroalkyl substances
PRM: parallel reaction monitoring
PTCR: proton transfer charge reduction
PTM: post‐translational modification
QqQ: triple quadrupole
REMPI: resonance‑enhanced multiphoton ionization
RF: radiofrequency
ROI: region(s) of interest
RTS: real‐time search
SICRIT: soft ionization by chemical reaction in transfer
SID: surface‐induced dissociation
SIM: selected ion monitoring
SLIM: structures for lossless ion manipulations
SPS: synchronous precursor selection
S/N: signal‑to‑noise
TMT: tandem mass tag(s)
TOF: time‐of‐flight
TRL: technology readiness level
UHPLC: ultra‑high performance liquid chromatography
UHV: ultra‑high vacuum
UVPD: ultraviolet photodissociation

## 1 Introduction

This year marks the 20th anniversary of Orbitrap™ Mass Spectrometry (OTMS) as a mainstream technology for high‑resolution, accurate‑mass (HR/AM) analysis. Anniversaries invite both celebration and reflection, and this review aims to examine where OTMS originated, where it stands today, and where it may develop in the future.

Two decades already constitute a meaningful historical timescale in our rapidly evolving world. It is therefore fitting to borrow an analytical framework from one of my hobbies—the study of civilizations.

“The Anna Karenina principle” was introduced by Jared Diamond (Diamond 1994) and later generalized in his book on human societies (Diamond 1997). The name of the principle derives from the famous first sentence of Tolstoy's great novel “Anna Karenina” (Tolstoy 2001): “Happy families are all alike; every unhappy family is unhappy in its own way.”

As I always struggled with understanding this sentence, Diamond's interpretation immediately caught my attention: for a marriage to succeed, a wide range of conditions must be simultaneously satisfied‐mutual attraction, agreement about money, child discipline, religion, in‐laws, and other vital issues. Failure in any one of those dimensions can doom the marriage even if it has all the other ingredients.

This concept generalizes well to many areas of life. People often seek simple, single‑factor explanations for success, but in reality, numerous potential failure points must all be avoided. On the pessimistic side, the principle implies that “no single property guarantees success, but many guarantee failure” (Diamond 1997).

Innovation follows a similar pattern: “Successful innovations **are all alike; every** failed innovation fails **in its own way**.” This principle comes as a very important complement to the known expression attributed to R. W. Emerson: “If you build a better mousetrap the world will beat a path to your door” (Emerson 1882). In reality, a breakthrough requires much more than a superior idea (Bornmann and Marx 2012). It demands a motivated and capable development team, supportive leadership, clear performance advantages, strong manufacturing practices, effective marketing, and the ability to sell and service the product.

All this was certainly true in the Orbitrap story. Each Orbitrap instrument got a good blend of all these factors, although each time slightly or considerably different. Most importantly, it has attracted and involved a lot of good and talented individuals. It is to these people that I dedicate this review of Orbitrap instrumentation!

Given this emphasis, the review focuses on innovations in instrumentation and the first demonstrations of new instruments, rather than attempting to cover the immense universe of Orbitrap applications. This universe keeps exponentially growing as reflected by a current publication rate exceeding 60 papers per day. Although broad reviews have appeared previously (Eliuk and Makarov 2015; Hecht et al. 2019; Deschamps et al. 2023), the field has grown beyond 160,000 Orbitrap‐based publications, making comprehensive coverage in a single review no longer feasible.

## 2 History Prior to the Commercial Launch

The long and complicated journey towards OTMS started around 30 years ago in a fertile land of blue‐sky ideas and discussions as vividly described in (Makarov 2013; Makarov 2019). This work was fueled by necessity to secure the future of the small startup HD Technologies Ltd that became my home, and by ambition to create a next‐generation mass analyzer that could combine all the strengths of the most advanced analyzers of the time such as Fourier transform Ion Cyclotron Resonance (FT ICR), time‐of‐flight (TOF) and radiofrequency (RF) ion traps (Brunée 1987).

In view of the unparalleled mass accuracy and resolution of FT ICR, image‑current detection was chosen as the fundamental operating principle. The large superconducting magnet, however, was replaced with a combination of dynamic ion trapping in Kingdon's electrostatic trap (Kingdon 1923; Knight 1981) and an electrostatic field known from energy analyzers Korsunskii and Basakutsa (1958) and TOF instruments (Gall et al. 1986). This refined geometry enabled axially harmonic orbital motion with oscillation frequency independent of ion energy and initial position (Makarov 1999).

Named the “Orbitrap mass analyzer” (Makarov 2000), the device utilized three electrodes—one central trapping electrode and two outer detection electrodes—and offered the highest oscillation frequency and best resistance to space‑charge effects for its size and voltage. Upon acquisition of HD Technologies in January 2000, Orbitrap became a trademark of Thermo Fisher Scientific Inc.

Maturation of this scientific curiosity into a practical Orbitrap analyzer required several more significant scientific and technological advances (Makarov et al. 2013). Among them were development and stable production of ultra‐precise Orbitrap electrodes, ultra‐stable high‐voltage power supplies, ultra‐high vacuum (UHV) systems and many others. The most crucial advance, however, was the separation of the ion source from the analyzer using an RF‑only storage device acting as a pseudo‑source (Makarov et al. 2002; Hardman and Makarov 2003; Hu et al. 2005). This ultimately resulted in the development of a novel device bent like a letter C and hence named the C‐trap (Makarov et al. 2005). It was interfaced to the electrostatic trap via novel, differentially pumped planar ion optics that also included vertical shift of ion trajectory to block the line of sight.

This new Orbitrap analyzer (Figure 1) was first introduced to a wider scientific community in 2005 as a part of the commercial LTQ Orbitrap hybrid instrument. This system married the sensitivity and ease of use of a linear ion trap with the ultra‑high resolution, mass accuracy, and dynamic range characteristic of FTMS with both analyzers operating in parallel (Makarov, Denisov, Kholomeev, et al. 2006). This launch opened access to this technology for the analytical community and therefore marked the entry of OTMS into mainstream mass spectrometry.

**Figure 1:**
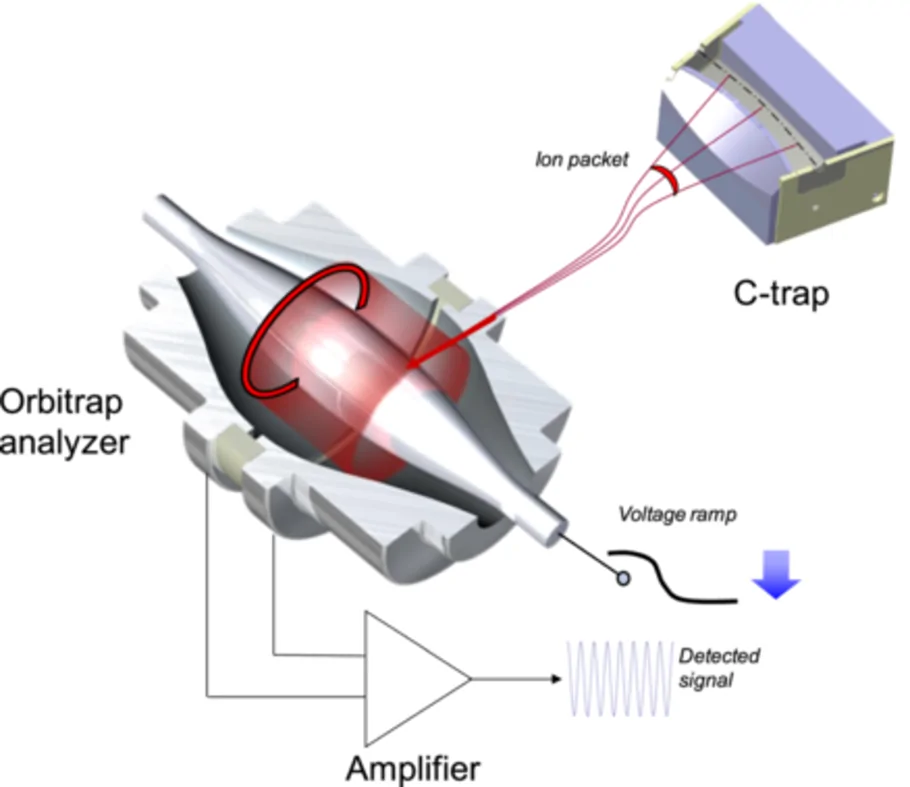
Schematic of the Orbitrap analyzer (ion optics and differential pumping not shown). Ions are injected into the RF‐only quadrupole of the C‐trap along its curved axis, where they undergo collisional cooling with bath gas and are accumulated along the axis, compressed toward the center by voltages applied to the end electrodes. At the beginning of each acquisition cycle, ions are released from the C‐trap as short packets by switching off the RF and applying a pulsed transverse electric field. These ion packets enter the analyzer while a voltage ramp is applied to the central electrode. During this ramp, the ions spread into rotating rings around the central electrode and are radially compressed until they reach an optimal orbit diameter midway between the central and outer electrodes. Because the ions are injected off the equatorial plane, the electrostatic field drives them toward the equator, initiating coherent axial oscillations along the central electrode. These axial oscillations induce image currents on the split outer electrodes, which are detected by a differential amplifier, digitized, and Fourier transformed to produce the frequency spectrum and the corresponding m/z spectrum. (Reproduced with permission of Thermo Fisher Scientific). [Color figure can be viewed at wileyonlinelibrary.com]

## 3 Technology Development of LTQ Orbitrap Family of Instruments

LTQ Orbitrap instrument experienced enthusiastic acceptance initially by proteomic (Olsen et al. 2005; Scigelova and Makarov 2006) and antidoping community (Thevis et al. 2005) as they were the first to appreciate the great gain in specificity enabled by superior Orbitrap mass accuracy, resolution and sensitivity in combination with MS^n^ capability. Other fields like Absorption, Distribution, Metabolism, Excretion, and Toxicity (ADMET) studies and metabolomics followed soon (Muenster and Makarov 2006, Lim et al. 2007; Scigelova and Makarov 2009). Typically, analysis was performed using Data‑dependent Acquisition (DDA), with overview Full MS spectrum acquired by Orbitrap analyzer and selected MS/MS spectra acquired using linear trap analyzer. This success created growing demand for further technology innovations that fueled growth of Orbitrap mass spectrometry. Figure 2 presents an overview of this growth over the entire 2 decades, as shown by the timeline on the left.

**Figure 2:**
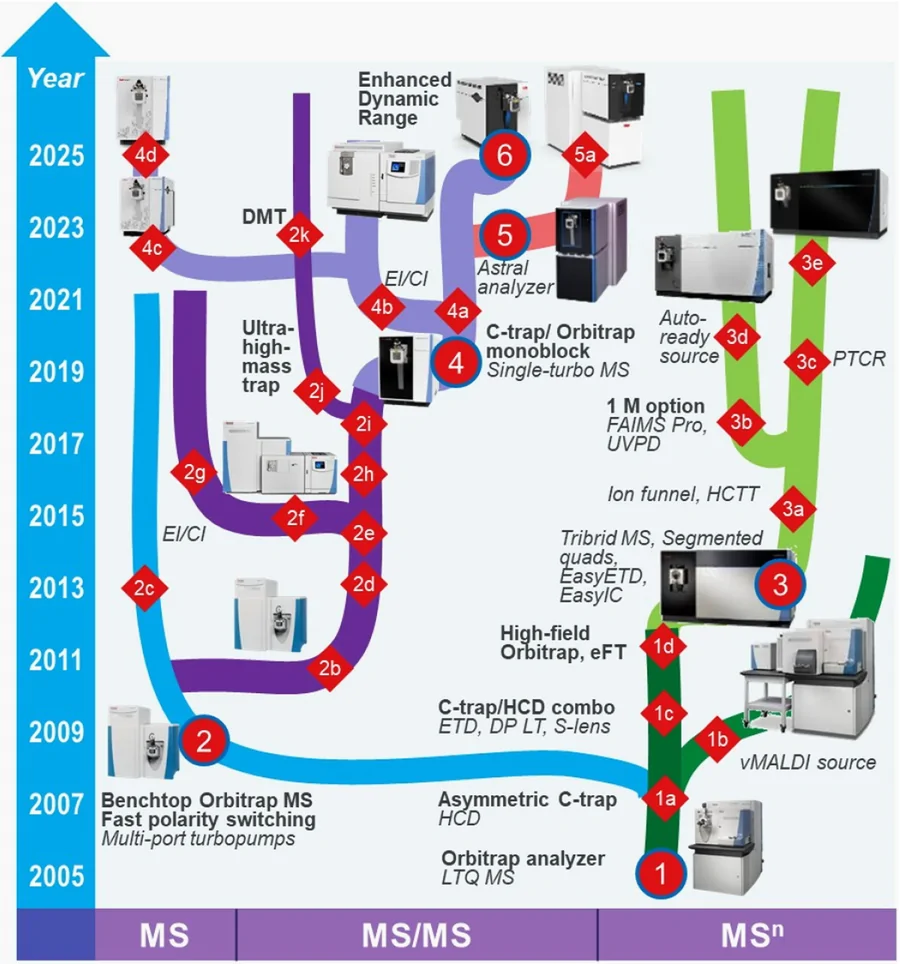
Evolution tree of the Orbitrap MS over the last two decades with time as the vertical dimension. The horizontal axis classifies instruments according to their analytical capabilities: MS‐only, MS^2^, and MS^n^. Key innovations in the Orbitrap analyzer itself are shown in **bold**, while developments in complementary technologies are indicated in *italics*. Instrument families are color‐coded, each red circle represents the start of a new instrument family while each diamond represents a subsequent product launch, sometimes encompassing multiple models: (1) LTQ Orbitrap MS followed by LTQ Orbitrap XL and Discovery (1a), LTQ Orbitrap MALDI (1b), Orbitrap Velos (1c), Orbitrap Elite and Velos Pro MS (1d). (2) Exactive MS followed by Q Exactive (2b), Exactive Plus (2c), Q Exactive Plus (2d), Q Exactive HF (2e), Q Exactive GC (2f), Exactive GC (2g), Q Exactive Focus (2h), Q Exactive HF‐X (2i), Q Exactive UHMR MS (2j), DMT option for Q Exactive UHMR MS (2k). (3) Orbitrap Fusion MS followed by Orbitrap Fusion Lumos (3a), Orbitrap ID‐X (3b) Orbitrap Eclipse (3c), Orbitrap IQ‐X (3d), Orbitrap Ascend MS (3e). (4) Orbitrap Exploris 480 MS followed by Orbitrap Exploris 120 and 240 (4a), Orbitrap Exploris GC and GC240 (4b), Orbitrap Exploris MX (4c), Orbitrap Exploris EFOX MS (4d). (5) Orbitrap Astral followed by Orbitrap Astral Zoom MS (5a). (6) Orbitrap Excedion Pro MS. (Reproduced with permission of Thermo Fisher Scientific). [Color figure can be viewed at wileyonlinelibrary.com]

The entire evolution tree is grouped according to analytical capabilities of the instruments, starting from MS‐only analysis, followed by MS/MS capabilities and finally MS^n^. Let us examine this development tree in greater detail in Sections 3, 4, 5, 6, 7, 8 as part of an exercise in instrumentation archaeology!

Following the launch of the LTQ Orbitrap MS in 2005, higher‐energy collisional dissociation (HCD) (Olsen et al. 2007) and the asymmetric C‐trap (Makarov et al. 2008) were introduced in 2007 as a part of LTQ Orbitrap XL MS. HCD in an RF‐only gas‐filled multipole enabled improved analysis of such important posttranslational modification (PTM) as phosphorylation. In parallel, a lower performance LTQ Orbitrap Discovery MS was launched, for the first time attempting to segment the market into low‐ and high‐tiers.

Behind these dry facts hides an emotional story of successes and failures in the Bremen factory. Unknown to the general public, the unexpected skyrocketing of demand exposed the lack of automation in instrument testing and reduction of Orbitrap manufacturing yield with increasing volumes, sometimes resulting in production stops to search for the root‐cause. This overloaded the small project team, with the pace of its growth not even closely following the expansion of work. This, in turn, has led to taking shortcuts during new product development carried out by the same team in parallel to production support. This revealed itself later, when a significant number of HCD cells had to be recalled in 2008 and replaced by a more elaborate and robust design.

In 2008, a dedicated LTQ Orbitrap instrument with Matrix‐Assisted Laser Desorption and Ionization (MALDI) ion source was unveiled (Strupat et al. 2009), featuring collisional cooling of MALDI ions. For many years it remained the ultimate MALDI imaging workhorse (Eliuk and Makarov 2015), offering a unique combination of high mass accuracy, resolution, dynamic range and MS^n^ capabilities. It also enabled, for the first time, the concept of acquiring a full mass spectrum from a single laser shot. It was also used for other applications, for example for biomass analysis using laser desorption (Smith and Lee 2010).

In 2009, an integrated C‐trap and HCD cell, back‐end electron transfer dissociation (ETD) (Syka et al. 2004; McAlister et al. 2008), dual pressure linear trap and a more sensitive atmosphere‐to‐vacuum interface became part of the LTQ Orbitrap Velos™ system (Olsen et al. 2009). The integrated C‐trap and HCD cell reduced ion losses on transfer and improved sensitivity, but most of the innovation came from the brand‐new LTQ Velos front end (Pekar Second et al. 2009) which also offered a back‐end ETD option that became indispensable for analysis of labile PTMs and proteins. Sensitivity was drastically increased by the introduction of a stacked‐ring ion guide (S‐lens), a simplified ion funnel without an axial field that uses a supersonic gas jet from a round‐bore transfer tube to propel ions through the lens.

There is no surprise that rapid switching of demand almost entirely to this new instrument brought some new challenges. While the S‐lens provided a 5–10× increase in ion current and thus sensitivity, it also led to a proportional acceleration of ion‐optics contamination. It turned out that there was simply not sufficient time during the development process to test all possible consequences of this effect. More importantly, the quality of some ion‐optical elements slightly deteriorated upon scaling up—stealthily enough not to be noticed during factory testing but clearly sufficient to cause charging after months of continuous operation under high sample load. This caused quite a lot of trouble for both the development and service teams as many instruments had to be upgraded later in the field with new solutions to reduce contamination.

As Orbitrap MS gained increased adoption, new applications continued to appear. One of the most important became analysis of intact proteins such as antibodies (Bondarenko et al. 2009). For the first time detection of individual multiply charged protein ions was demonstrated (Makarov and Denisov 2009), paving the way to Orbitrap charge‐detection MS (CDMS) of the 2020s.

Having LTQ mass spectrometer as the front‐end, all instruments of this family also were naturally able to operate with different front‐end options as described in Section 10.

The pinnacle of the first‐generation Orbitrap family became the Orbitrap Elite™ instrument (Figure 3) (Michalski, Damoc, Lange, et al. 2011). It incorporated two key innovations, first being a novel high‐field Orbitrap design as shown on Figure 4 alongside the original version. It almost doubled the acquisition speed and increased sensitivity. Another key innovation, enhanced Fourier transform (eFT™) processing combined so‐called absorption mode with magnitude mode and provided another doubling of resolving power (Lange et al. 2014), Overall, this last and ultimate member of the LTQ Orbitrap family quadrupled resolution per unit time compared to previous instruments. It also incorporated a significantly higher‐temperature bakeout of the Orbitrap analyzer, enabling improved ultra‐high vacuum conditions and thereby facilitating its increasing use in top‐down protein analysis (Fornelli et al. 2012). This marked the beginning of a long journey toward subsequent breakthroughs in high‐mass Orbitrap analysis.

**Figure 3:**
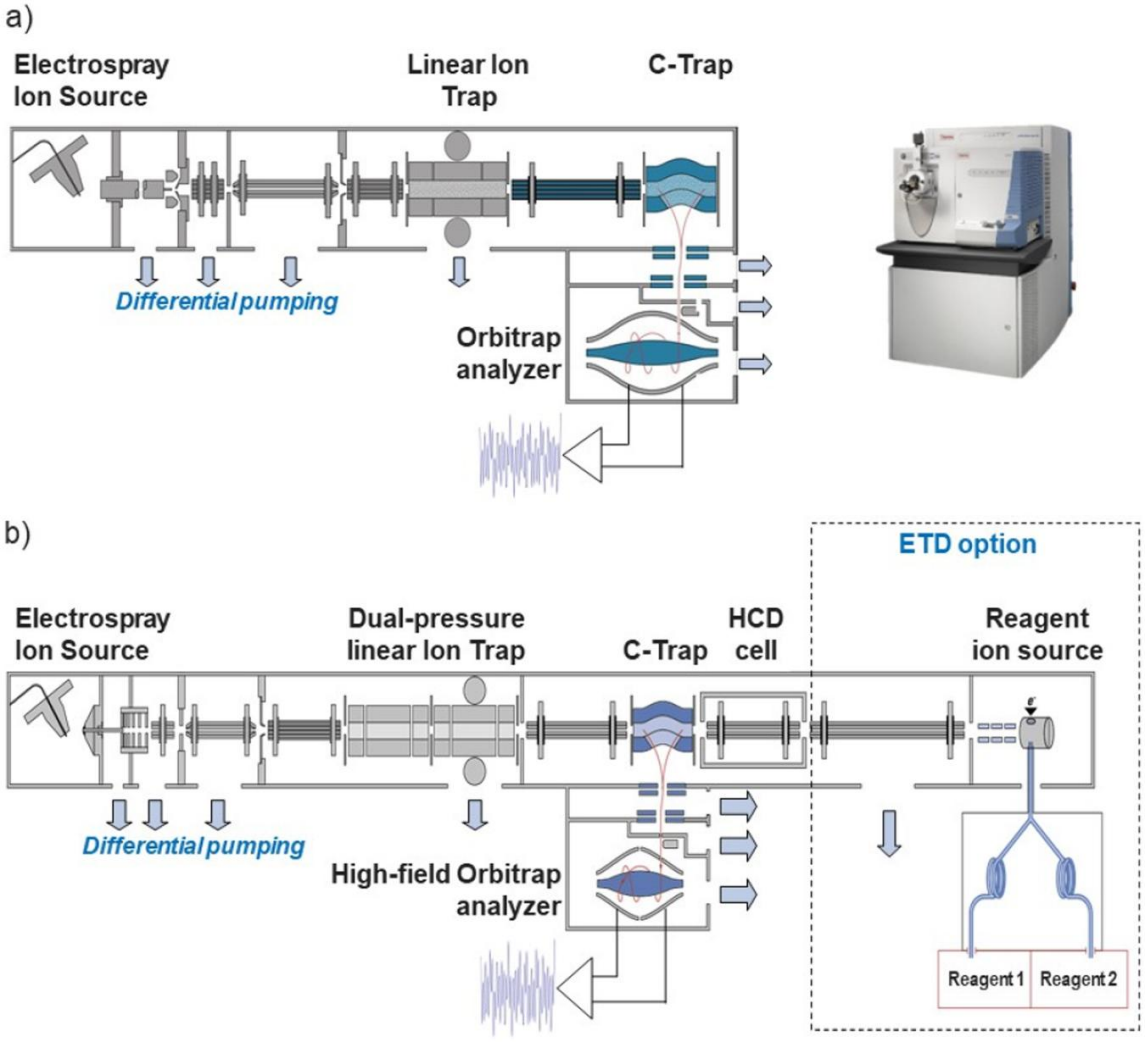
Schematic layout and picture of a linear trap‐Orbitrap hybrid MS: (a) LTQ Orbitrap MS, (b) Orbitrap Elite MS with ETD option (reproduced with permission of Thermo Fisher Scientific). [Color figure can be viewed at wileyonlinelibrary.com]

**Figure 4:**
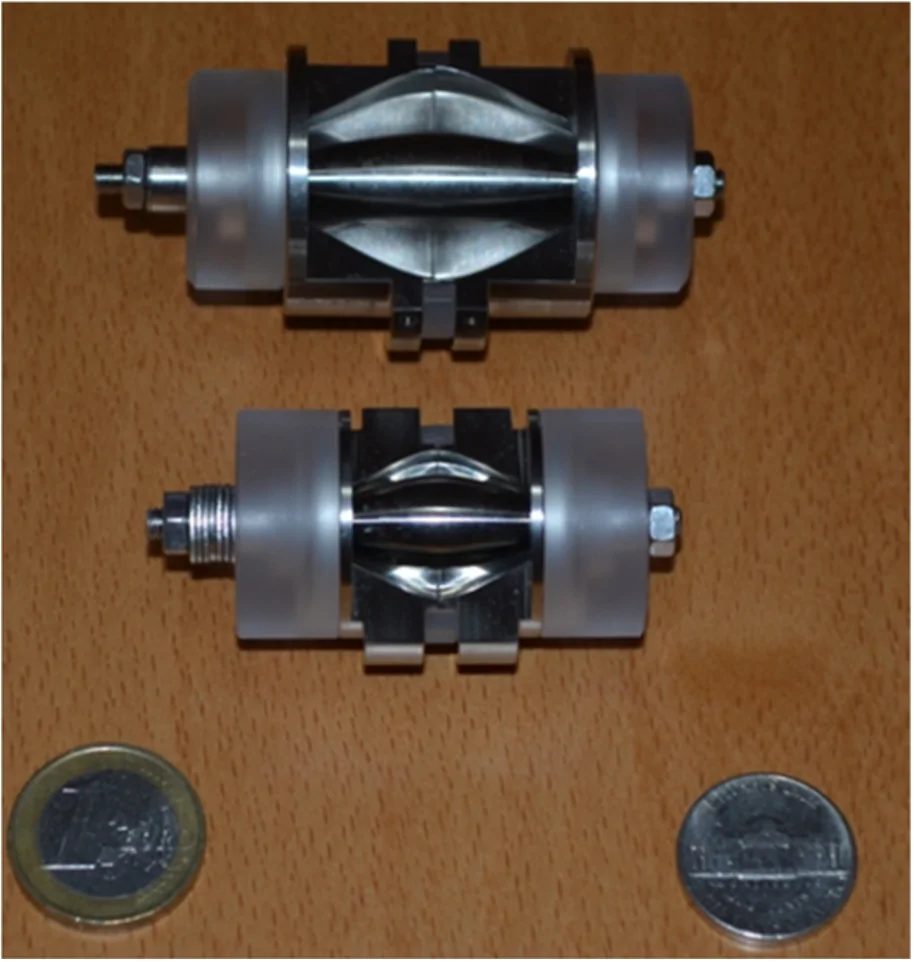
Cut‐outs of a standard (top) and a high‐field (bottom) Orbitrap analyzer (reproduced with permission of Thermo Fisher Scientific). [Color figure can be viewed at wileyonlinelibrary.com]

## 4 Tribrid Family of Instruments as the Next Generation MS^n^ System

A major breakthrough along the MS^n^ line came in 2013 with the launch of the Orbitrap Fusion™ Tribrid™ MS (Senko et al. 2013). Here a high‐field Orbitrap analyzer was combined with a segmented hyperbolic quadrupole mass filter and a dual‐pressure linear trap (Figure 5). Based on lessons learned from the LTQ Orbitrap platform, the Orbitrap analyzer in this instrument was moved to precede the linear trap, with HCD cell extending its role to a bi‐directional ion transfer and becoming an Ion Routing Multipole (IRM). The Orbitrap operating voltage was increased from 3.5 kV in Orbitrap Elite MS to 5 kV, further increasing the speed of analysis for a given resolution.

**Figure 5:**
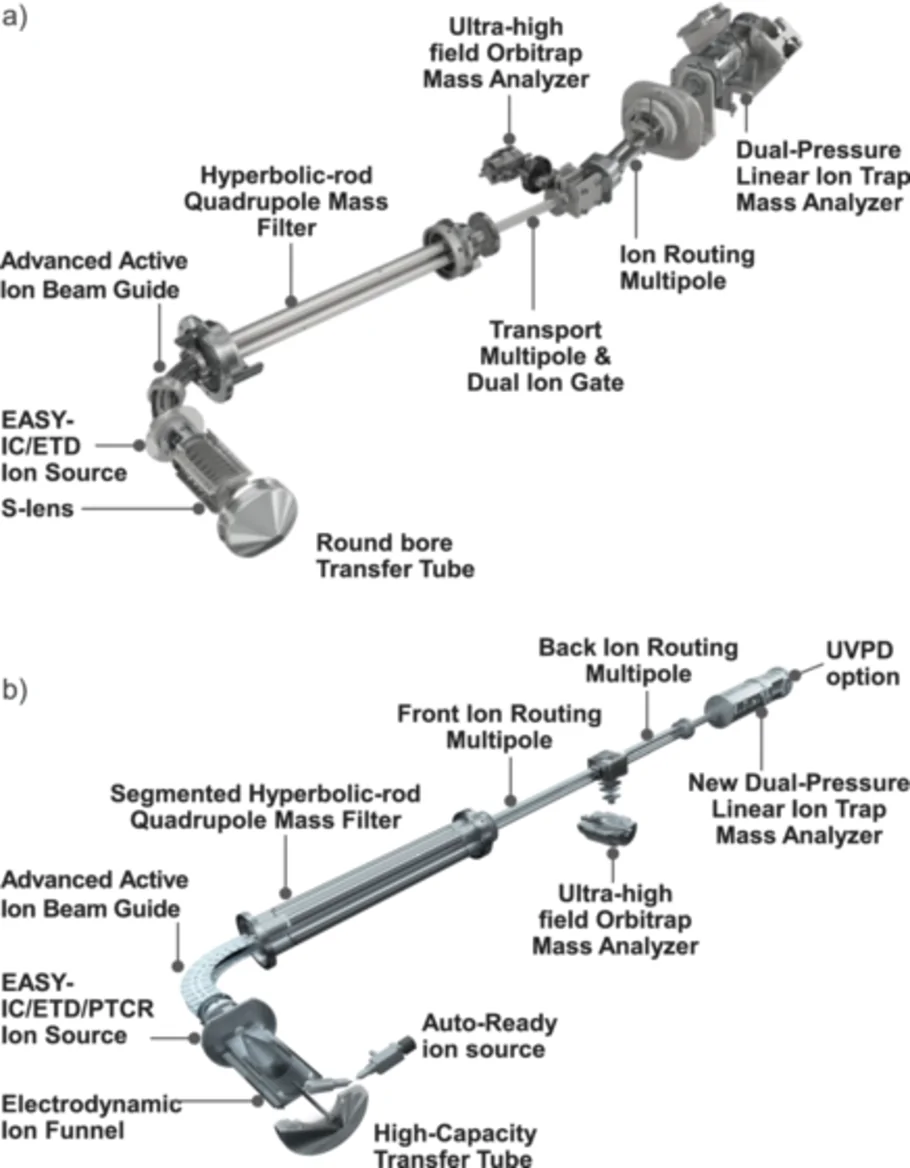
Schematic layout of a Tribrid MS: (a) Orbitrap Fusion and Orbitrap ID‐X MS; (b) Orbitrap Ascend MS (reproduced with permission of Thermo Fisher Scientific). [Color figure can be viewed at wileyonlinelibrary.com]

This instrument also featured front‐end internal calibration source (Easy‐IC™ source) that could be also used for ETD (Easy‐ETD™) fragmentation. To maximize peptide/protein sequence coverage, ETD could be also combined with CID in the linear trap to yield so‐called ETciD as well as with HCD in the IRM to result in EThcD (Frese et al. 2012). This in turn enabled new applications such as top‐down sequencing (Fornelli et al. 2017) and discrimination of leucine and isoleucine in peptide sequencing (Lebedev et al. 2014).

Improved instrument control enabled truly parallel operation of all three analyzers (dynamic scan management) and new modes of operation, such as Synchronous Precursor Selection (SPS) by multinotch isolation in the linear trap. This mode became the basis for Tandem Mass Tag (TMT) MS^3^ workflow to overcome the issue of ratio compression (McAlister et al. 2014).

In 2015, Orbitrap Fusion Lumos™ MS pioneered the next generation atmosphere‐to‐vacuum interface. There the round‐bore transfer tube and S‐lens of Orbitrap Fusion MS was superseded by a high‐capacity transfer tube (HCTT) of slit‐like cross‐section and ion funnel. Resulting 4x increase in incoming gas flow became the decisive factor for the dramatic increase of sensitivity. The instrument also featured more elaborate pressure control in IRM, improving top‐down analysis.

Since 2017, Tribrid instruments became capable to support options of 2nd generation Field Asymmetric Waveform Ion Mobility Spectrometry (FAIMS Pro™ system) (Pfammatter et al. 2018; Melani et al. 2019; Goswami et al. 2025), Ultra‐Violet Photo‐Dissociation (UVPD) (Fornelli et al. 2020) and 1 Million mass resolution (1 M) (Denisov et al. 2012).

In 2018, a variant of the original Orbitrap Fusion was launched as Orbitrap ID‐X™ MS for small molecule analysis. It has introduced AcquireX™ data acquisition mode for automated iterative DDA analysis in metabolome profiling.

Orbitrap Eclipse™ became the next highest performance Orbitrap MS in 2019. The arsenal of fragmentation methods was further enhanced by adding Proton Transfer Charge Reduction (PTCR) using negatively charged reagent in Easy‐ETD (Huguet et al. 2019). This method became an increasingly powerful method for untangling complexity of overlapping multiply charged fragments.

Larger diameter hyperbolic‐rod quadrupole enabled narrower mass filtering and higher resistance to contamination from the deposited ion beam.

TMT and other DDA methods were improved by Real‐Time Search (RTS), in which on‐the‐fly spectral searching allowed to save time on spectra of insufficient quality (Erickson et al. 2019). Specifically for TMT, a new phase‐constrained signal deconvolution method (FSDM) (Grinfeld et al. 2017) was applied to double the speed of reporter ion measurement.

The increase of mass range of isolation and analysis has enabled the innovative method of NativeOmics, wherein MS^n^ of protein complexes made it possible for the first time to sequence also endogenous lipids deep inside those complexes (Gault et al. 2020).

The next‐generation instrument for small molecule analysis, the Orbitrap IQ‐X™ MS, was introduced in 2021. Developed on the Orbitrap Fusion Lumos platform, it featured an Auto‐Ready ion source that allows fully automated calibration using a parallel source independent of the LC inlet.

Orbitrap Ascend MS was launched in 2022, and it signified a drastic redesign of the instrument architecture, adding an additional IRM in front of the C‐trap as a buffer for ion storage prior to MS^n^ experiments and an Auto‐Ready ion source for automatic system calibration. The mass range of linear trap isolation was extended to 8000 m/z and Orbitrap detection‐ to 16000 m/z. The instrument also used a longer ion funnel to improve analysis of labile compounds, from phosphopeptides to metabolites (He et al. 2023).

Since 2024, this very versatile model has been offered in three fit‐for‐purpose Editions such as multiomics, structural biology and biopharma. For the latter two, quadrupole electronics was reworked to enable high‐mass isolation up to 8000 m/z, further extending instrument applications in Native MS. New methods such as DIA‐PTCR for top‐down mass spectrometry allowed to detect up to an order of magnitude more proteoforms (Schachner et al. 2024).

This ever‐ongoing Tribrid evolution enables analysis of analytes and complex mixtures which even recently have been beyond the reach of mass spectrometry as witnessed by deep analysis of membrane protein complexes (Lutomski et al. 2025; Schachner et al. 2024).

## 5 (Q) Exactive Family of Instruments as the First Milestone in Democratizing HR/AM Analysis

### 5.1 Toward Democratization of HR/AM

Although Orbitrap MS rather unexpectedly became the top tier of mass‐spectrometric instrumentation and, by the late 2000s, largely displaced the previous crown bearer FT ICR MS, it was always clear to us that the true destiny of Orbitrap technology was the democratization of HR/AM analysis.

For this reason, already 1 year after the launch of the very first LTQ Orbitrap instrument, active research began along two very different paths: the first—to make Orbitrap‐based instrumentation more affordable and easier to use, and the second—to develop an analyzer that could eventually surpass the Orbitrap as the next performance leader.

The first direction led to the (Q) Exactive™ and later the Orbitrap Exploris™ and Excedion™ product lines, while the second ultimately resulted in the emergence of the Astral™ platform in 2023.

Naturally, the first direction received the highest priority because market demand for accessible HR/AM instruments already existed. However, addressing this need required a complete redesign of the earlier Orbitrap technology. In the run‐up to the LTQ Orbitrap launch, so many aspects of the system were still unknown that even reasonably predictable components (e.g., differential pumping, thermal isolation and stabilization, ultra‐high vacuum sealings) were deliberately over‐engineered to avoid surprises during production. As we embarked on designing a cost‐efficient Orbitrap instrument, we had to re‐work and optimize the entire instrument layout.

It became apparent that the new instrument had to be benchtop, and that dedicated multistage turbopumps would be required to replace the numerous pumps used in the LTQ Orbitrap MS. The first instrument in the Exactive series included only the Orbitrap analyzer for MS‐only operation, although its mechanical layout intentionally left sufficient room for the later addition of a quadrupole mass filter. The main challenges were largely engineering‐related: we needed to demonstrate that the Orbitrap analyzer could operate reliably in an aluminum—rather than a stainless‐steel chamber, without ConFlat seals and without water‐cooling for temperature stabilization and bakeout, and that it could still detect ions in the presence of numerous electrical and mechanical interferences inside a tightly packed benchtop enclosure. These challenges were successfully resolved by a small R&D team working closely with vacuum pump vendors, although several unexpected side effects of the new layout forced us to abandon a few configurations as technological dead ends. Later, as instrument production scaled up, numerous new issues with electronic boards emerged and required multiple redesigns—changes that eventually proved indispensable for the ramp‐up to the next‐generation Orbitrap hybrids.

### 5.2 Main Instruments of the Q Exactive LC Family

The Exactive MS was announced as early as 2008, and shipments began in 2009 (Bateman et al. 2009) (Figure 2). In the absence of LTQ detection, it relied on Orbitrap detection for Automatic Gain Control (AGC) (Stafford et al. 1992), that is, to regulate the filling time of the C‐trap. Because the instrument acquired only full‐scan MS spectra without mass selection (Figure 6), its sole option for structural elucidation was All‐Ion Fragmentation (AIF) (Geiger et al. 2010).

**Figure 6:**
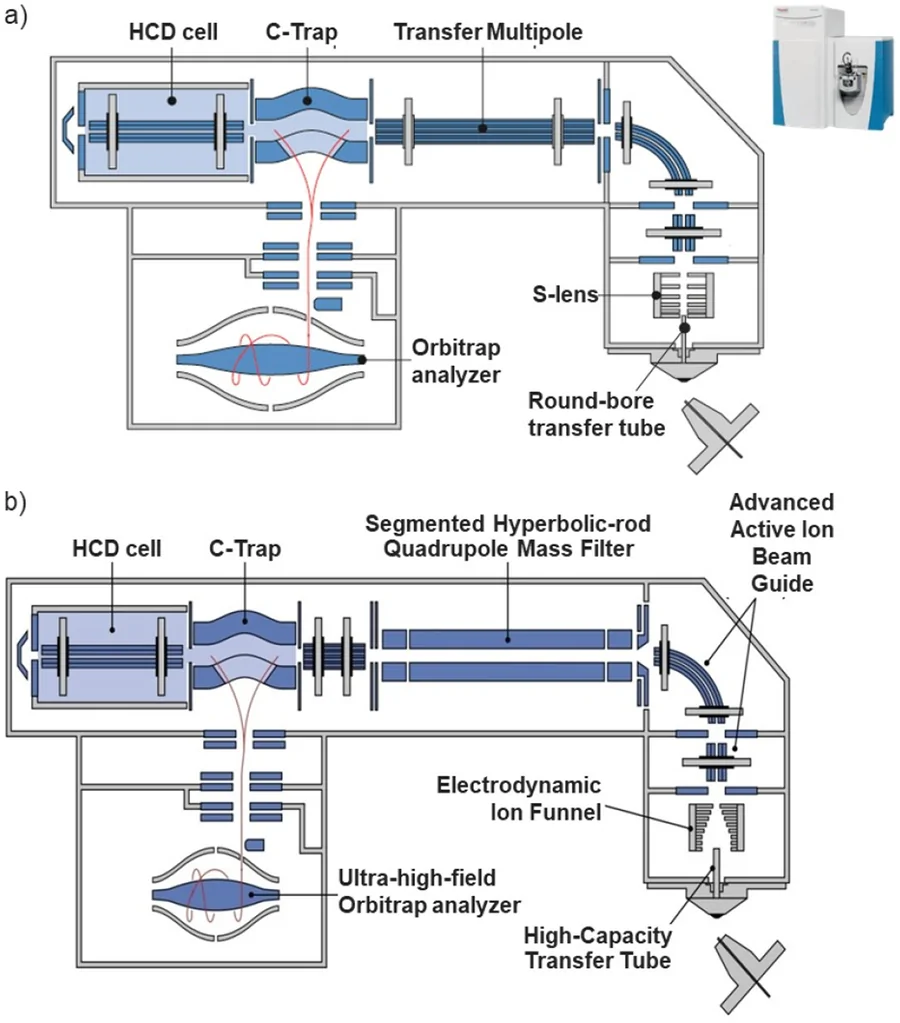
Schematic layout of (a) a stand‐alone Orbitrap instrument, the Exactive Plus MS; (b) quadrupole‐Orbitrap hybrid mass spectrometer, the Q Exactive MS (reproduced with permission of Thermo Fisher Scientific). [Color figure can be viewed at wileyonlinelibrary.com]

This instrument took advantage of having just a single ultra‐precise voltage to run the analyzer: as this voltage needed switching on every ion injection, it was now switched continuously between −5 and +5 kV power supplies, both being ultra‐precise and temperature‐stabilized via Peltier elements. Keeping both supplies constantly “warm” meant that polarity could be alternated from one scan to the next simply by adjusting the switching timing, without sacrificing part‐per‐million external mass accuracy. This unique capability became widely adopted in various small‐molecule screening applications (Moulard et al. 2011).

The next‐generation instrument, launched in 2011 in parallel with Orbitrap Elite MS, was the Q Exactive quadrupole–Orbitrap hybrid (Michalski, Damoc, Hauschild, et al. 2011). To ensure efficient filling of MS2 spectra, AGC was enhanced by adding an independent ion‐current measurement device in the form of an electrometer located after the HCD cell. This allowed to include into the fill time prediction, a substantial part of ion current in complex mixtures that fell below detection threshold in the Orbitrap spectra. Externally, the instrument closely resembled the Exactive, and it soon became a routine workhorse in thousands of laboratories worldwide.

Three years later, a dedicated edition of the Q Exactive MS for environmental, food safety, and toxicology applications was introduced as the Q Exactive Focus MS (Simirgiotis et al. 2016; Wang et al. 2017). Meanwhile, the Exactive MS itself was upgraded and released as the Exactive Plus MS (Rochat et al. 2012; Berube et al. 2012).

As the Q Exactive platform proved both more affordable and more productive in DDA experiments than the LTQ Orbitrap family (Kelstrup et al. 2012), it rapidly became a true workhorse for HR/AM analysis. This widespread adoption drove continual performance improvements. In 2013, the Q Exactive Plus MS introduced an improved hyperbolic quadrupole mass filter with pre‐ and postfilters, preceded by a new ion guide that utilized Printed Circuit Boards (PCBs) to generate an axial field. This configuration doubled MS/MS sensitivity and substantially extended intervals between required cleanings when analyzing complex “‐omics” mixtures. The instrument also offered higher mass resolution and an intact‐protein mode.

In 2014, the family was further extended with an ultra‐high‐field Orbitrap analyzer, similar to the one used in the Tribrid series, resulting in the Q Exactive HF MS (Scheltema et al. 2014). Then, in 2017, the system was further enhanced with a high‐capacity transfer tube and an ion funnel design similar to those on the Orbitrap Fusion Lumos MS, yielding the Q Exactive HF‐X MS (Kelstrup, Bekker‐Jensen, et al. 2018). These instruments were optimized for fast separations by Ultra‑high Performance Liquid Chromatography (UHPLC) as well as for new methods of data‐independent acquisition (DIA) (Panchaud et al. 2009) and parallel reaction monitoring (PRM) (Peterson et al. 2012). Both DIA and PRM relied on pushing the limits of high resolution and accurate mass of panoramic MS/MS spectra.

This parade of product names and milestones may give the impression of an uninterrupted march of success, but it does not adequately reflect the enormous amount of work behind the scenes—across R&D, marketing, product support, and the global sales organization. Although the democratization of HR/MS was ultimately successful, progress was repeatedly challenged by unexpected complications arising from the “chaotic” side of Mother Nature and the inherent complexity of high‐tech manufacturing.

Two particularly devastating incidents are especially memorable. The first resulted from a small, unannounced cost‐saving change introduced by a turbopump vendor in the rotor bearing design—after which UHV pumps began failing after only 1–2 years of operation. It took years to identify and correct the root cause across thousands of instruments worldwide, delivering a significant reputational blow to the brand.

The second incident arose from a similarly unannounced modification made by the supplier of the C‐trap end‐lenses in response to tightening environmental regulations. Metal‐coated ceramic lenses that had functioned flawlessly since the early LTQ Orbitrap days began to exhibit microscopic metallization defects, which charged up after weeks of continuous exposure to the intense ion beams in the Q Exactive HF‐X MS. Because this effect was easily masked by other potential sources of charging, it proved exceptionally difficult to trace and ultimately required the recall of many tens of instruments, followed by extensive field upgrades to repair the remaining systems.

Looking back, we would have spared no effort to avoid these failures, particularly in light of the substantial damage and disruption they caused for our valued and trusting users and their research!

### 5.3 Orbitrap MS for High‐Mass Analysis

As understanding of the motion of intact proteins within the Orbitrap analyzer deepened (Scigelova and Makarov 2009), Orbitrap instruments continued to expand their accessible mass range toward increasingly large macromolecular assemblies. A major breakthrough emerged through collaboration with the Prof. Heck's group (Rose et al. 2012; Rosati et al. 2012), which demonstrated for the first time high‐sensitivity, high‐resolution native analysis of large intact protein complexes such as GroEL. This work led to the development of the Exactive EMR instrument series, featuring Orbitrap detection extended beyond 20,000 m/z (Maple et al. 2014).

Subsequent improvements focused on widening the mass range of quadrupole isolation (Belov et al. 2013; Gault et al. 2016; Dyachenko et al. 2015), extending the mass range of the ion optics by employing lower‐frequency RF waveforms (Snijder et al. 2014), and introducing in‐source trapping to enhance desolvation and fragmentation (Van de Waterbeemd et al. 2017; Fort, Van de Waterbeemd, et al. 2018). These advancements culminated in the 2018 release of the Q Exactive UHMR MS, the final instrument in the mighty Q Exactive family.

High‐mass analysis was further advanced with the introduction of individual‐ion MS signal processing approaches (Kafader, Melani, et al. 2019; Kafader, Beu, et al. 2019; Kafader et al. 2020; Wörner et al. 2020), which were incorporated in 2022 into the Direct Mass Technology (DMT) option (Strasser et al. 2023). This represented the first commercial implementation of Charge Detection Mass Spectrometry (CDMS), previously available only in research‐grade instruments (Fuerstenau and Benner 1995; Contino et al. 2013). Compared with the linear electrostatic traps traditionally used for CDMS, the Orbitrap analyzer offers exceptionally high space‐charge capacity and an ion frequency that is independent of ion energy, enabling simultaneous CDMS measurement of hundreds, and sometimes thousands, of individual ions within a single acquisition.

## 6 Exploris and Excedion Instruments Supersede the Q Exactive Hybrid Family

### 6.1 Next Step in Democratization of HR/AM

Building on the experience gained with the Q Exactive series, it became possible to commit to a new ambitious goal of making the hybrid quadrupole–Orbitrap architecture as amenable to mass production and broad analytical utilization as the most mass‐produced tandem mass spectrometers, that is, triple quadrupoles. This effort evolved into the Orbitrap Exploris MS platform, guided by the following design principles (Hauschild et al. 2020):

- More efficient use of physical space and improved serviceability, achieved largely by reducing the size of the pumping system and ion optics. A novel six‐port turbopump was a key innovation, enabling a two‐fold reduction in instrument size. Initially deemed impossible, such a pump became the first stone in the foundation of the Exploris platform, with first experiments starting as early as 2010 at Pfeiffer Vacuum GmbH.
- Performance flexibility enabled by modular hardware and software components, with increased commonality shared with Tribrid instruments. This work began in earnest in the mid‐2010 s as Tribrid platform started to mature.
- Greater use of electronics‐inspired technologies in ion‐optical design, particularly the implementation of PCB‐based RF ion guides.
- Harmonization of interfaces across instrument families, facilitating workflow sharing and easier transfer of analytical methods. Because it uses the same ion source as the Tribrid MS platform, the Exploris family supports the same options, including the FAIMS Pro interface and the EASY‐IC internal lock‐mass ion source.

The C‐trap and Orbitrap analyzer were merged into a single monobloc assembly to improve robustness against noise and mechanical misalignment, with symmetrically supported Orbitrap electrodes providing enhanced noise cancellation.

An independent charge detector (ICD) module was placed upstream of the C‐trap to increase AGC speed and to free the rear of the HCD cell for future extensions, effectively transforming it into an ion‐routing multipole similar to that used in Tribrid systems. This design choice later proved crucial for interfacing the Exploris platform with the Astral back end.

In almost every field of hardware development, establishment of a new platform is associated with a much higher effort, risk and overall uncertainty than any, however ambitious, extension of an existing platform. This appeared to be especially true for the Exploris platform as development took longer than expected and priorities of development were adjusted “on the go” in response to changes in business priorities.

### 6.2 Main Instruments of Next‐Generation Orbitrap Hybrids

The high‐end Orbitrap Exploris 480 MS, featuring an HCTT/ion‐funnel interface (Figure 7), was launched in 2019 for *‐omics* applications [Bekker‐Jensen et al. 2020]. Its model number reflects the maximum resolving‐power setting. Like the Orbitrap Eclipse MS, it incorporated the FSDM method, which doubles the speed of TMT reporter‐ion analysis (Grinfeld et al. 2017; Kelstrup, Aizikov, et al. 2018). With the Biopharma option, it is optimized for implementing different variants of multi‑attribute method (MAM) in biopharmaceutical analysis.

**Figure 7:**
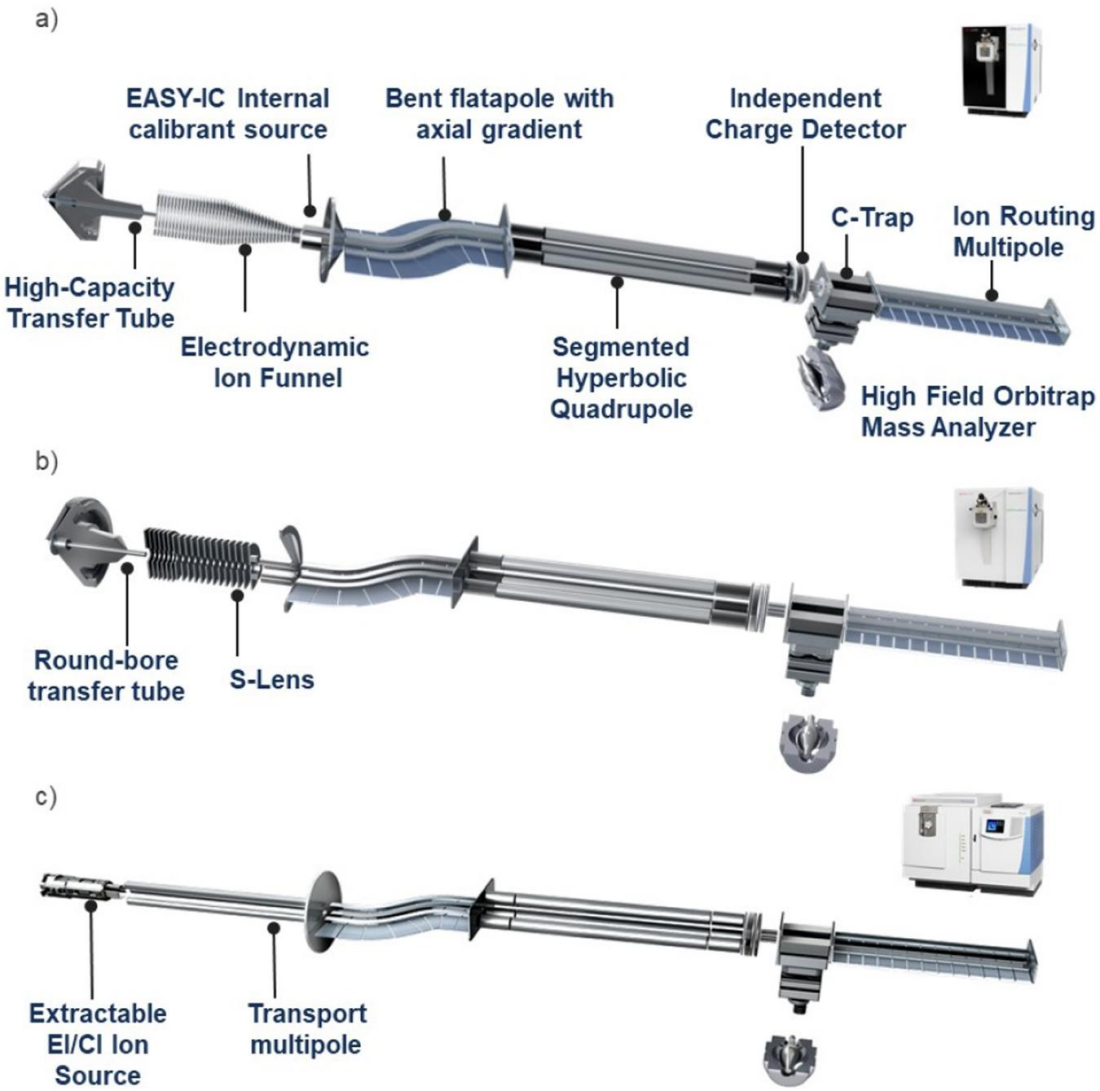
Schematic layout of an Orbitrap Exploris hybrid MS: (a) Orbitrap Exploris 480 MS, (b) Orbitrap Exploris 120 and 240 MS, (c) Orbitrap Exploris GC and GC 240 MS (reproduced with permission of Thermo Fisher Scientific). [Color figure can be viewed at wileyonlinelibrary.com]

The following year, the universal Orbitrap Exploris 240 MS was introduced (Knizner et al. 2022), together with the Orbitrap Exploris 120 MS (Monaghan et al. 2021). These instruments featured a more traditional round‐bore transfer tube/S‐lens interface and maximum resolution settings reflected by the model number. They were optimized for quantitative environmental, food‐safety, toxicology, and other small‐molecule applications. This successful dual launch, at the height of the COVID‐19 lockdown, was made possible only by the timely implementation of a comprehensive remote instrument control system in the preceding years.

In 2022, the product line was complemented by the MS‐only Orbitrap Exploris MX MS, designed specifically for quality control in biopharmaceutical manufacturing. Another version of this MS‐only instrument was launched in 2025 for environmental, forensic and toxicology analysis under the name Orbitrap Exploris EFOX MS.

In 2025, the Orbitrap Exploris 480 MS was superseded by the Orbitrap Excedion Pro MS. This model added a more robust sprayer, a gentler ion‐funnel interface similar to that used in the Orbitrap Ascend MS, and—most importantly—Easy‐ETD capability together with EThcD support (Kessler et al. 2025). It also introduced the Enhanced Dynamic Range (EDR) Full‐MS scanning mode for the Orbitrap analyzer, extending the method first evaluated in (Meier et al. 2018).

### 6.3 Isotope Ratio Measurements

When issues with isotope accuracy were identified in early Orbitrap spectra in the late 2000s (Kaufmann and Walker 2012), dedicated internal research was initiated and accompanied by continuous improvements in Orbitrap hardware quality and signal‐processing methods. As accuracy improved, a productive collaboration was established with Prof. Eiler's group at Caltech, aiming to achieve the pro‐mille‐level isotope‐ratio precision required for the majority of applications. This goal was eventually met for intact organic molecules analyzed using both Gas Chromatography (GC) (Eiler et al. 2017; Hofmann et al. 2020; Zeichner et al. 2022) and LC separations (Mueller et al. 2021).

Building on this success, the Orbitrap Exploris Isotope Solutions package was introduced in 2022. It enables direct measurement and calculation of isotope ratios on any Exploris MS from the relative abundances of Isotopologues in an intact compound or its fragments (Hilkert et al. 2021). The solution includes a dual‐inlet unit and a flow‐injection valve, opening a new field of application for Orbitrap MS in isotope‐ratio measurement—an area previously dominated by magnetic‐sector instruments with multiple collectors.

Independently of this work, collaboration with Prof. Zubarev's group at the Karolinska Institut led to the development of a proteomics‐compatible method for isotopic‐ratio determination based on immonium fragments with baseline‐resolved isotopologues (Gharibi, Chernobrovkin, Saei, et al. 2022; Gharibi, Chernobrovkin, Eriksson, et al. 2022).

## 7 Astral Analyzer Enhances and Complements Orbitrap Performance

The ASymmetric TRAck Lossless (Astral) analyzer (Stewart et al. 2023) represents the culmination of many years of development that began in parallel with the Exactive series of instruments. Its most distinctive feature is a multi‐reflection analyzer formed by two opposing planar mirrors that are tilted relative to one another (Figure 8), with an ion foil positioned between them (Grinfeld et al. 2024). Ions from a quasi‐continuous beam are stored—and, if desired, fragmented—in an ion processor derived from the C‐trap but redesigned to provide much tighter spatial and temporal focusing (Stewart, Grinfeld, Wagner, et al. 2024). When ions are pulsed into the mirror assembly through prism‐based ion optics, the combined action of the mirror tilt and the ion foil reverses the ion trajectories as they bounce repeatedly between the mirrors, ultimately focusing them onto a novel high‐dynamic‐range detector. The entire system is gridless and delivers exceptionally high‐fidelity spatial and temporal focusing, making it effectively “lossless,” with ion losses far lower than those observed in any previous mass analyzer.

**Figure 8:**
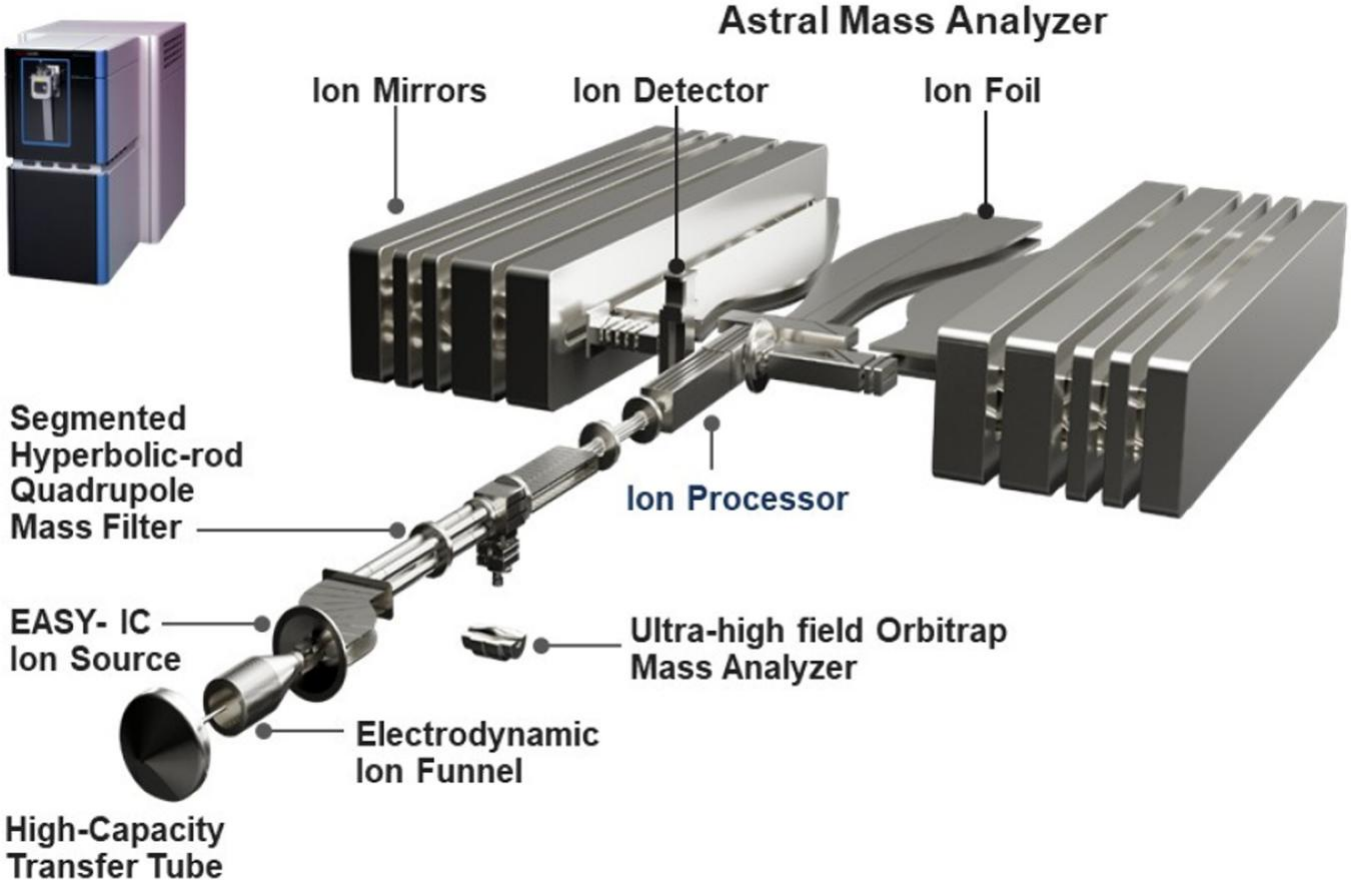
Schematic layout of an Orbitrap Astral MS (reproduced with permission of Thermo Fisher Scientific). [Color figure can be viewed at wileyonlinelibrary.com]

The Astral analyzer achieves single‐ion sensitivity at mass resolutions up to 100,000 and ppm‐level mass accuracy, while operating at acquisition speeds that initially reached 200 spectra per second and now extend up to 270 spectra per second. Each spectrum is fully compatible with modern data‐analysis workflows, as it typically contains thousands of ions.

In the Orbitrap Astral MS, the complementary strengths of the Orbitrap and Astral analyzers are exploited to their fullest extent. The Orbitrap analyzer, offering the highest ultimate resolution and external mass accuracy, is dedicated to Full MS acquisition, whereas the Astral analyzer—with its extraordinary speed and sensitivity—is used for MS/MS spectra. Together, they form an almost ideal envelope of analyzer performance, a synergy reflected in the numerous groundbreaking achievements reported in *‐omics* research (Guzman et al. 2024; Serrano et al. 2024; Lancaster et al. 2024; Petrosius et al. 2025].

In 2025 the second model, Orbitrap Astral Zoom MS (Hsu et al. 2025) was launched, offering higher acquisition rate and multipass operation for even higher resolution (Stewart, Grinfeld, Giannakopulos, et al. 2024). It is entirely plausible that, over time, the Astral analyzer may diverge from the Orbitrap lineage and establish a lineage of its own!

## 8 10 Years of Orbitrap GC/MS

The 20th anniversary of Orbitrap MS coincides with another important milestone: the 10‐year anniversary of the first serial GC–Orbitrap product. As in many developments, this instrument emerged from a highly productive collaboration—this time with Prof. Coon's group at the University of Wisconsin–Madison. While developing a back‐end ETD option for the LTQ Orbitrap MS that made use of a standard electron impact (EI) source from a GC–quadrupole instrument (McAlister et al. 2008), the group revealed an opportunity to apply this configuration directly to conventional GC analytes (Peterson et al. 2010). The concept was later extended to the Q Exactive instrument (Peterson et al. 2014), demonstrating the need for the newest high‐field Orbitrap analyzer to secure a sufficient number of data points across a characteristically narrow GC peak.

Building on this foundation, the first GC/Q‐Exactive instrument with EI and CI ion sources was introduced in 2015 (Weidt et al. 2016), followed a year later by a simpler MS‐only GC/Exactive model. The combination of very high sensitivity, mass accuracy, and resolution enabled detection limits that were comparable even in Full‐MS mode to those achieved with triple‐quadrupole (QqQ) instruments operating in their most sensitive single‐reaction‐monitoring (SRM) mode (Belarbi et al. 2021).

As the (Q) Exactive family was succeeded by the Exploris line, the GC‐Orbitrap platform followed the same evolution. In 2021, the earlier instruments were replaced by the MS‐only Orbitrap Exploris GC and the hybrid Orbitrap Exploris 240 GC, which now serve as the next‐generation GC–Orbitrap systems.

It is noteworthy that GC/MS was the first field to see the entry of a new vendor, Anyeep, in 2024 (Suzhou Anyi Spectrum 2025), with the so‐called Cassitrap analyzer, which matches the standard Orbitrap geometry.

## 9 Understanding the Orbitrap Analyzer

Ultra‐high resolving power in the Orbitrap analyzer became achievable in practice only when a deeper understanding of nonlinear, space‐charge–dominated ion oscillations in a perturbed quadratic potential was combined with manufacturing techniques capable of producing electrode geometries with accuracies in the high‐nanometer range.

Although ion motion in the Orbitrap field appears to follow the simple equations of 18th‐century celestial mechanics (Makarov 2000), real ion packets exhibit far more complex behavior, requiring analytical tools from 21st century physics of complex systems (Bolotskikh et al. 2011; Kharchenko et al. 2012; Skoblin et al. 2015). It was found that space‐charge interactions between ion packets depend strongly on even slight deviations of the electric field from ideality, leading to phenomena such as coalescence of adjacent intense peaks (Gorshkov et al. 2012; Kaufmann and Walker 2018; Grinfeld et al. 2019). Optimal performance is achieved when the trap incorporates a specific micrometer‐scale nonlinearity in its field profile (Grinfeld et al. 2015; Grinfeld et al. 2019). As space charge pushes ion rings to larger oscillation amplitudes, carefully shaping the field in these outer regions compensates for the expansion, enabling linear corrections to space‐charge influence on ion frequency.

Space‐charge effects are even more pronounced in the C‐trap, as shown by both simulations (Grinfeld et al. 2011) and experimental studies (Gorshkov et al. 2010; Kaufmann et al. 2010). These effects can be mitigated through intelligent C‐trap filling strategies (Kaufmann and Walker 2016; Meier et al. 2018; Panepinto et al. 2025), ultimately culminating in the EDR scan introduced with the Orbitrap Excedion Pro MS.

As manufacturing tolerances advanced into the high‐nanometer regime, progressively higher resolving powers could be achieved at increasingly high coalescence thresholds—first up to one million (Denisov et al. 2012), and later to two million and beyond (Denisov et al. 2021; Deslignière et al. 2024). This progress has allowed Orbitrap MS to expand into essentially all application areas historically addressed by FT ICR MS, such as petroleomics (Zhurov et al. 2013; Schmidt et al. 2018).

Although the Orbitrap analyzer is typically used solely for the detection of injected ions, several additional operating modes are also possible. As demonstrated in (Perry et al. 2008) and references therein, the analyzer can support high‐fidelity de‐excitation and re‐excitation of specific ions. While still largely unexplored, such modes may become valuable in the future, possibly for the analysis of very large macromolecular assemblies reaching the so‐called “plateau of stability” (Wörner et al. 2022).

Although the Orbitrap analyzer represents a specific axially symmetric realization of the broader class of Cassini fields (Köster 2009), its rotational symmetry makes it much simpler and therefore much more practical compared to other members of this field's family. Compared with other electrostatic trap geometries, such as linear or toroidal traps (Makarov 2010), the Orbitrap design provides the highest oscillation frequency—and therefore the highest resolving power for a given transient duration—at a given size and ion energy. Although signal processing is often regarded as independent from hardware development, in Orbitrap MS the two are deeply intertwined. One of the key factors is the importance of a virtual common starting point for ion trajectories as a side effect of rapid extraction from the C‐trap. This allows the initial phase for each m/z to be precisely defined and calibrated, enabling a two‐fold increase in resolution via enhanced Fourier transform (eFT) processing (Lange et al. 2014) and a further doubling beyond the Fourier limit through FSDM (Grinfeld et al. 2017; Kelstrup, Aizikov, et al. 2018; Steigerwald et al. 2024). Both methods also feature improved peak shape when dead time at the beginning of the transient is minimized. This required a refinement of preamplifier electronics and replacement of dielectric Orbitrap spacers to reduce dielectric losses. Signal processing of spectra with significant Coulomb interactions or with high in‐spectrum dynamic range has led to the development of sophisticated models for Orbitrap noise (Keenan et al. 2025).

Dedicated hardware and software from Spectroswiss (Nagornov et al. 2020; Leach et al. 2024) have enabled the user community to access raw transients directly and experiment with alternative signal‐processing methods. These tools also allow commercial instruments to operate in special modes that extend transient durations beyond standard settings (Nagornov et al. 2020), supporting experiments with ultra‐long transients (Deslignière et al. 2024). When ion masses within a narrow range are known *a priori*, least‐squares fitting can further improve resolving power (Aushev et al. 2014; Kozhinov et al. 2023).

Collision cross‐sections (CCS) of analyte ions can be extracted directly from Orbitrap transients alongside m/z by analyzing the exponential decay of ion signals caused by gas‐phase collisions within the analyzer. Early work (Sanders et al. 2018) established the fundamental “hard‐sphere” model linking transient damping to CCS. Subsequent studies refined the data‐processing framework, extended applicability to native proteins, and introduced “soft‐sphere” or energy‐dependent models that improve accuracy for low‐charge or low‐energy ions (James et al. 2022; James et al. 2023). The method now applies to analytes ranging from small molecules (Ni and Arevalo 2024) to large proteins, with good correlation to low‐field ion‐mobility CCS measurements when pressure and ion energy are well controlled and calibration against an internal standard is used. Current efforts aim to extend CCS determination to individual‐ion signal processing.

## 10 Orbitrap MS Outside of Mainstream

### 10.1 Introduction

As mainstream commercial Orbitrap MS evolved into a powerful evolution tree, an entire Amazon jungle of different third party solutions sprouted around it. Many of these developments introduced alternative approaches to ion generation and fragmentation, tailored to specific applications and market needs. With their number now reaching into the hundreds, this review necessarily concentrates on those options and extensions that have had the greatest impact or achieved broad recognition. Regrettably, the sheer diversity of these contributions precludes detailed treatment of each. Instead, a more structured, taxonomic overview is adopted—an approach that can only partially capture the richness and creativity of this highly dynamic and diverse landscape, which ultimately reflects the remarkable versatility of mass spectrometry as an analytical technique.

### 10.2 Front‐End Options and Ion Sources

Already the very first Orbitrap MS family benefited from a wide range of plug‐in commercial front‐end technologies originally developed for LTQ MS including:

- Field Asymmetric waveform Ion Mobility Spectrometry (FAIMS) (Saba et al. 2009) from Thermo Fisher Scientific,
- Atmospheric pressure (AP) matrix assisted laser desorption and ionization (MALDI) source (McEwen et al. 2010), commercialized by Masstech Inc.
- Desorption electrospray ionization (DESI) [(Hu et al. 2006), commercialized by Prosolia Inc.
- Direct analysis in real time (DART) (Romão et al. 2016), commercialized by IonSense Inc.
- Laser diode thermal desorption (LDTD) (Munoz et al. 2015) from Phytronix Technologies Inc.
- Atmospheric pressure photo ionization (APPI) (Núñez et al. 2012) from Syagen Inc.
- Advion nanomate for high‐throughput infusion experiments, for example in lipidomics (Schuhmann et al. 2012).

These ion sources interfaced directly with the standard electrospray hardware port of the instrument.

Later, additional plug‐in ion sources became commercially available for hybrid and Tribrid Orbitrap systems, such as:

- Atmospheric solids analysis probe (ASAP) (McEwen and Gutteridge 2007), (Jagerdeo and Wriston 2017) later commercialized by ASAP-MS.com
- APPI GC interface developed in collaboration with Prof. Benter's group at the Wuppertal University and later commercialized by Mascom GmbH (Kersten et al. 2016).
- Soft ionization by chemical reaction in transfer (SICRIT) (Basham et al. 2022)
- Multiple ionization methods under the umbrella of “inlet ionization” (Trimpin et al. 2024), originating from (McEwen et al. 2010, Pagnotti et al. 2011) and later commercialized by MSTM Inc.
- High‐resolution atmospheric pressure (AP) MALDI from Transmit GmbH (Kompauer et al. 2017).
- Multisprayer chip by Newomics Inc. (Chen et al. 2018).
- Multi‐scheme chemical ionization (MION) by Karsa Oy (Partovi et al. 2023).
- Secondary electrospray ionization source for breath analysis commercialized by Fossilion (Singh et al. 2018, Singh et al. 2019) following initial research in Zenobi's group (García‐Gómez et al. 2015, Gaugg et al. 2016).
- Laser ablation electrospray ionization DP‐1000 source marketed for several years by Protea Biosciences (Beach et al. 2016). Some plug‐in sources remain research‐grade and used by multiple groups in custom‐built configurations, although not yet commercialized:
- Liquid sampling–atmospheric pressure glow discharge (LS‐APGD) for combined atomic and molecular ionization (Quarles et al. 2012, Kenneth Marcus 2023).
- MassSpec Pen ambient sampling device for in‐surgery diagnostics (Zhang et al. 2017).
- SpiderMass source for ambient analysis of biological tissues ablated by a fiber laser (Ogrinc et al. 2019).
- AP matrix‐assisted laser desorption electrospray ionization (MALDESI) (Barry et al. 2014).
- nanoDESI (Roach et al. 2010).
- Photoinduced thermal desorption on an atomic force microscope platform coupled with mass spectrometry for multimodal imaging (Lorenz et al. 2019).

These lists are almost certainly incomplete, as numerous research groups worldwide continue developing specialized setups for specific applications.

A number of unique ion sources require deep modifications to the Orbitrap instrument itself, including:

- Dual ESI/MALDI (Belov et al. 2017) and MALDI‐2 (Niehaus et al. 2019) sources operating at sub‐mbar pressures, marketed by Spectroglyph LLC.
- Liquid injection field desorption ionization (LIFDI) (Braunschweig et al. 2017; Légaré et al. 2018) commercialized by Linden CMS GmbH
- ConDuct interface for increasing ion transmission from atmospheric pressure (Krutchinsky et al. 2015).
- Resonance‐enhanced multiphoton ionization (REMPI) with ionization inside the C‐trap (Kösling et al. 2021).
- Inductively coupled plasma (ICP) source (described only in conference reports)
- Direct laser ionization in vacuum without using the C‐trap from a surface, either without (Makarov et al. 2013) or with matrix assistance (Grechnikov et al. 2014).

The following subsections describe techniques and applications that required similarly extensive modifications to Orbitrap instruments.

### 10.3 OrbiSIMS MS

The commercialization of the OrbiSIMS instrument by IonTOF GmbH represents one of the most successful examples of adapting Orbitrap MS to a completely new type of analysis. In OrbiSIMS, secondary ions are generated by bombardment of a flat sample with primary Bi‐ or Ar‐cluster ion beams under high‐vacuum conditions (Passarelli et al. 2017). The Orbitrap analyzer provides high‐resolution MS and MS/MS, complementing a fast but lower‐resolution TOF analyzer.

The concerted operation of both analyzers enables imaging at both macroscopic and microscopic (submicron) scales, while delivering excellent mass resolution and MS/MS sensitivity for selected regions of interest (ROI).

Such instruments have become powerful tools for biological analysis (Zhang et al. 2020) as well as for the study of organic‐based microelectronic devices such as organic light‐emitting diodes (OLED) circuits (Trindade et al. 2023).

### 10.4 Ion Mobility on Orbitrap MS

As ion mobility expanded rapidly over the past 25 years, numerous efforts were made to interface different ion‐mobility techniques with Orbitrap MS despite substantial differences in their timescales of separation. In addition to FAIMS option described above, several notable implementations include:

- LC‐compatible atmospheric‐pressure ion mobility spectrometry (IMS) drift tube with single and double gating (Hagan et al. 2017) by Excellims,
- AP drift tube (Keelor et al. 2017) developed by Prof. Clowers,
- Low‐pressure drift tube (Ibrahim et al. 2016) with signal averaging, single or double multiplexing IMS.
- Fourier transform ion mobility‐orbitrap mass spectrometer for native mass spectrometry (Poltash et al. 2018).
- Structures for lossless ion manipulations (SLIM) (Hollerbach et al. 2024; Deng et al. 2025).
- High‐resolution planar FAIMS (Baird et al. 2019), later commercialized by Heartland Mobility LLC.

Although the relatively long (10s–100s ms) cycle time of image‐current detection in Orbitrap MS is not well suited to the millisecond‐scale peaks produced by conventional drift tubes, ion mobility information may nevertheless be retained, for example, through the use of linked‐scan approaches as described in (Makarov 2025).

### 10.5 Light Spectroscopy and Photofragmentation

The trapping nature of Orbitrap instruments makes them naturally well suited for photofragmentation, for example, in a RF‐only collision cell. Also, it enables even light‐based spectroscopy across a wide range of wavelengths, from vacuum ultraviolet to the far‐infrared ends of the spectrum.

Infrared multiphoton dissociation (IRMPD) was the first photofragmentation technique applied to Orbitrap MS (Vasicek et al. 2011). It was later used to improve peptide sequence coverage by providing supplemental activation for ETD (Riley et al. 2017), and it has also been applied to the desolvation and sequencing of large protein complexes (Lutomski et al. 2023).

UVPD was initially implemented using excimer lasers (Shaw et al. 2013), first for top‐down protein analysis and later for a broad spectrum of applications, ranging from lipids (O'Brien et al. 2014) to large protein complexes under native conditions (Fort et al. 2016; Brodbelt et al. 2019). UVPD using solid‐state lasers was subsequently introduced (Halim et al. 2015) and became the basis for the commercial UVPD option on Orbitrap Tribrid MS systems (Fornelli et al. 2020).

While many groups implemented fixed‐wavelength photofragmentation across different parts of the spectrum (Brodbelt et al. 2019), far fewer were able to pursue wavelength‐scanning approaches. This latter strategy enabled action spectroscopy (Kopysov et al. 2015; Kopysov et al. 2016; Zviagin et al. 2025), in which fragment‐ion spectra reflect the internal structure of the trapped ions. Because fragmentation efficiency depends strongly on chromophore environment, the method provides remarkable structural specificity, enabling separation and quantification of even co‐eluting isomers of the same molecule. Its applicability has been extended to weakly absorbing species such as lipids by adding fluorophores that form complexes with the analytes (Saparbaev et al. 2021).

### 10.6 Orbitrap Analyzer for Space Research

As LTQ Orbitrap MS was applied to laboratory analogues of Titan aerosols (Pernot et al. 2010; Gautier et al. 2014), it became evident that the Orbitrap analyzer held significant potential for space research‐particularly for missions involving in‐situ sampling of extraterrestrial materials (Cottin et al. 2010). This realization led to the formation of the CosmOrbitrap consortium, which launched a long‐term effort to develop lightweight, low‐power Orbitrap‐based instruments and progressively raise their Technology Readiness Level (TRL) (Briois et al. 2016; Arevalo et al. 2018).

Current work focuses on the most pragmatic aspect of space exploration: the analysis of lunar surface materials for minerals and organics to support future lunar habitats (Willhite et al. 2020). A long‐term strategic aim is the eventual deployment of Orbitrap analyzers for in‐situ astrobiology missions, enabling the high‐resolution characterization of complex organic signatures beyond Earth (Arevalo et al. 2023).

### 10.7 Emerging Fragmentation Techniques Integrated With Orbitrap Analysis

The past decade has brought significant advances in fragmentation technologies which turned out to be synergetic with the high resolution and sensitivity of Orbitrap MS.

It has been demonstrated that positively charged biological ions can be activated using beams of high‐energy cations (Chingin et al. 2014). The Electron‐Capture Dissociation (ECD) option developed by eMSion Inc. has proven valuable for protein fragmentation (Shaw et al. 2018; Fort, Cramer, et al. 2018) and for charge reduction of large protein complexes up to intact viruses (Le Huray et al. 2024; Peris‐Díaz et al. 2025).

Surface‐induced dissociation (SID) (VanAernum et al. 2019) has emerged as a powerful method for analyzing noncovalent complexes of biological significance, with Orbitrap detection enabling improved quantification of ligand binding and resolution of complex isotopic envelopes.

Combining Orbitrap MS with the Omnitrap platform (Papanastasiou et al. 2022) represents another step toward “freedom of fragmentation.” This configuration enables a combinatorial explosion of MS^n^ fragmentation schemes, spanning a wide range of electron energies and optionally incorporating IR and UV lasers (Smyrnakis et al. 2023). Notably, recent work showed that only distinct narrow ranges of electron energies improve sequence coverage most effectively. In another set of bottom‐up experiments, fine‐tuned electron energies enabled detailed sequencing of biantennary glycopeptides (Li et al. 2024) and even singly charged glycans (Wei et al. 2023). The utility of Omnitrap–Orbitrap integration for top‐down MS was further demonstrated for an endogenous antibody light chain (Garcia et al. 2025).

### 10.8 Preparative Orbitrap MS

Recent years have seen the rise of a powerful new emerging application for the long‐established technique of soft‐landing ions onto surfaces: in‐vacuo preparation of samples for cryogenic electron microscopy (CryoEM).

Initial demonstrations by Prof. S. Rauschenbach's group (Fremdling et al. 2022; Esser et al. 2022) were later complemented by work from Prof. J. Coon, who focused on depositing ions onto a matrix (Westphall et al. 2022; Salome et al. 2022; Lee et al. 2023). Embedding ions in ice (Esser et al. 2022) under cryogenic conditions (Westphall et al. 2023) proved instrumental for improving CryoEM resolution (Esser et al. 2024). Furthermore, laser re‐melting has shown promise for restoring the native structure of protein complexes after deposition (Mertz et al. 2025), enabling accurate determination of their 3D structures by CryoEM.

Soft‐landing setup have also shown utility for depositing analytes onto other types of sensors, such as nanoelectromechanical systems (Neumann et al. 2024).

### 10.9 “Paper Instruments”

A plethora of Orbitrap‐based instrument concepts has been proposed—typically, in patents—but still remain on paper for various practical reasons:

- Multielectrode Orbitrap devices such as (Makarov 2007, Li and Mordehai 2010) offer dynamic voltage control across multiple electrodes with simpler geometries such as circular rings. However, they still require the same ultra‐high mechanical precision as conventional Orbitrap electrodes, making fabrication and alignment probably even more challenging due to their quantity and the number of very accurate voltage supplies.
- Orbitrap instruments with “slow” injection (Makarov 1999; Doroshenko and Misharin 2014; Makarov 2017): while avoiding fast RF switching, this approach requires broadband excitation similar to FT‐ICR and introduces additional cycle‐time overhead, significantly reducing its practical attractiveness.
- Multiple detection electrodes (Makarov, Denisov, Jung, et al. 2006; Makarov 2012, Jarrold and Todd 2022) could enable detection higher‐frequency and hence higher throughput detection (e.g., via enhanced third harmonics of the axial frequency) or improved sensitivity by detecting image current on both inner and outer electrodes. In practice, however, implementation is difficult due to requirements for extremely small gaps, ultra‐high geometric precision, and/or floating ultra‐sensitive preamplifiers at time‐dependent high voltages.
- Multi‐Orbitrap design (Makarov and Horning 2008) allows to increase throughput of analysis roughly proportionally to the number of analyzers operating in parallel. However, this benefit comes with nearly linear increases in mechanical and electronic complexity and cost, with only modest savings from shared vacuum systems and power supplies. Notably, the value of operating two high‐resolution analyzers in parallel has since been demonstrated in the Orbitrap Astral platform, where one Orbitrap performs slower ultra‐high‐resolution acquisition (potentially including EDR), while the second analyzer focuses exclusively on high‐speed MS/MS acquisition. Overall, each of these concepts is fundamentally feasible, and some may yet prove valuable as enabling technologies and engineering solutions continue to evolve.

## 11 Future of Orbitrap MS

With the Astral analyzer now representing the current pinnacle of performance due to its superior acquisition rate and sensitivity, Orbitrap technology is ready to become the major workhorse of high‐resolution mass spectrometry. This evolution is expected to unfold along several major directions.

The first major thrust will involve the democratization of Orbitrap technology toward mass‐detector–class instruments, as already demonstrated with the Orbitrap Exploris MX and EFOX systems. This trend is anticipated to extend further into mobile and field‐deployable platforms, including applications such as intraoperative analysis with the MassSpec Pen.

The second major thrust involves the deeper integration of Orbitrap analyzers with complementary technologies, including:

- New instrument configurations, continuing the trend seen in Tribrid, Orbitrap Astral, OrbiSIMS MS.
- Integration with emerging fragmentation methods, particularly those involving electrons and photons.
- Support for non‐MS modalities such as ion mobility, spectroscopy, preparative MS, and so forth.
- Deployment within specialized instruments for clinical diagnostics, isotope‐ratio measurements, structural biology, de novo sequencing, per‐/poly‐fluoroalkyl substances (PFAS) analysis, and more
- Fit‐for‐purpose interfaces to specialized ion sources, such as ICP and photoionization sources

Both of these trends share the focus on cost‐optimized mechanical design, contamination‐resistant ion optics, remote and online diagnostics, and automatic calibration.

In addition, numerous opportunities remain for improving Orbitrap performance. A major avenue is the extension of DMT to new applications across both higher and lower m/z ranges, enabled by advances in signal‐processing techniques.

New Orbitrap designs could further enhance performance—whether optimized specifically for DMT or implemented in multitrap architectures (Makarov and Horning 2008). Ultra‐long transients offer the potential to reach extreme resolving powers in the multimillion range.

Further improvements in operational methods are expected as well. For example, EDR scanning may continue to increase the depth and dynamic range of screening workflows.

Novel signal‐processing methods such as FSDM (Kelstrup, Aizikov, et al. 2018; Steigerwald et al. 2024) can double resolving power for the same transient duration, while transient‐fitting approaches hold promise for improving isotope‐ratio measurements, structural analysis, and CCS determinations (James et al. 2022; James et al. 2023; Sanders et al. 2018).

As Orbitrap technology matures, future advances are likely to arise less from purely hardware‐driven improvements and increasingly from the integration of sophisticated software and artificial intelligence (AI).

Intelligent control algorithms can enable adaptive data acquisition, dynamically optimizing resolution, ion injection, and fragmentation methods in real time. AI‐enhanced signal processing may further improve effective resolution, mass accuracy, intensity accuracy and dynamic range by mitigating noise and space‐charge effects. In parallel, predictive diagnostics and automated calibration can enhance robustness and facilitate remote, autonomous operation.

Ultimately, the integration of high‐fidelity hardware with intelligent software layers may shift Orbitrap mass spectrometry toward becoming an integral part of self‐optimizing, experiment‐adaptive analytical systems. As a part of such integration, it is conceivable that for certain well‐defined and routine analytical tasks, simpler configurations (e.g., MS‐only platforms) could replace more complex tandem MS systems.

## 12 Conclusion

Orbitrap mass spectrometry has advanced enormously over the past 20 years and continues to evolve. During this period, the performance frontiers of Orbitrap technology have expanded enormously across nearly every analytical dimension. Limits of quantitation below 1 attomole, acquisition speeds reaching 70 spectra per second, mass resolutions exceeding 2 million, isotope‐ratio precision better than 0.1%, and sub‐ppm mass accuracy have become hallmarks of the technology, enabling highly confident molecular identification.

Orbitrap technology also continues to redefine the boundaries of protein analysis. The latest instruments can isotopically resolve intact antibodies and determine the charge states of individual ions across an exceptionally broad mass range, from small peptides to ions with masses in the multi‐megadalton regime.

This level of performance has led to widespread global deployment, with a five‐digit number of Orbitrap instruments operating worldwide around the clock across nearly all domains of mass spectrometry, contributing to the scientific literature at a rate of two to three publications per hour.

By continuing to advance Orbitrap technology and integrating it more deeply into analytical workflows, our goal is to bring high‐resolution mass spectrometry to every laboratory, ultimately contributing to a safer, cleaner, and healthier world!

## Author Contributions

**Alexander Makarov:** conceptualization, writing – original draft, writing – review and editing, investigation, visualization.
